# Evolution of Neuroplasticity in Response to Physical Activity in Old Age: The Case for Dancing

**DOI:** 10.3389/fnagi.2017.00056

**Published:** 2017-03-14

**Authors:** Patrick Müller, Kathrin Rehfeld, Marlen Schmicker, Anita Hökelmann, Milos Dordevic, Volkmar Lessmann, Tanja Brigadski, Jörn Kaufmann, Notger G. Müller

**Affiliations:** ^1^Neuroprotection Laboratory, German Center for Neurodegenerative Diseases (DZNE)Magdeburg, Germany; ^2^Institute of Sport Science, Otto-von-Guericke-UniversityMagdeburg, Germany; ^3^Medical Faculty, Institute of Physiology, Otto-von-Guericke UniversityMagdeburg, Germany; ^4^Center for Behavioral Brain Sciences (CBBS)Magdeburg, Germany; ^5^Informatics and Microsystem Technology, University of Applied Science KaiserslauternKaiserslautern, Germany; ^6^Medical Faculty, Clinic for Neurology, Otto-von-Guericke UniversityMagdeburg, Germany

**Keywords:** neuroplasticity, VBM, exercise, dancing, neurodegeneration, BDNF

## Abstract

From animal research, it is known that combining physical activity with sensory enrichment has stronger and longer-lasting effects on the brain than either treatment alone. For humans dancing has been suggested to be analogous to such combined training. Here we assessed whether a newly designed dance training program that stresses the constant learning of new movement patterns is superior in terms of neuroplasticity to conventional fitness activities with repetitive exercises and whether extending the training duration has additional benefits. Twenty-two healthy seniors (63–80 years) who had been randomly assigned to either a dance or a sport group completed the entire 18-month study. MRI, BDNF and neuropsychological tests were performed at baseline and after 6 and 18 months of intervention. After 6 months, we found a significant increase in gray matter volume in the left precentral gyrus in the dancers compared to controls. This neuroplasticity effect may have been mediated by the increased BDNF plasma levels observed in the dancers. Regarding cognitive measures, both groups showed significant improvements in attention after 6 months and in verbal memory after 18 months. In addition, volume increases in the parahippocampal region were observed in the dancers after 18 months. The results of our study suggest that participating in a long-term dance program that requires constant cognitive and motor learning is superior to engaging in repetitive physical exercises in inducing neuroplasticity in the brains of seniors. Therefore, dance is highly promising in its potential to counteract age-related gray matter decline.

## Introduction

The current demographic change in Western societies, involving both a relative and an absolute increase in the number of older people, has sparked increasing scientific interest in geriatric issues. In this context, concepts of successful aging are becoming increasingly important. Several studies have indicated that physical exercise may play a key role in healthy aging and in the prevention of cognitive decline and neurodegenerative diseases (Colcombe et al., 2006; Erickson et al., 2011). Recent reviews summarizing epidemiological, cross-sectional and interventional studies support physical activity as a propitious method to induce neuroplasticity in late adulthood (Gregory et al., 2012; Voelcker-Rehage and Niemann, 2013; Bamadis et al., 2014). Erickson et al. (2012) have concluded that higher cardiorespiratory fitness and aerobic physical activity levels are associated with larger gray matter volumes in the prefrontal regions and the hippocampus. However, not only cardiovascular fitness but also coordinative exercise (Niemann et al., 2014) and cognitive training (Bamadis et al., 2014) have been shown to induce gray matter plasticity and enhance cognitive functions in older adults.

Animal research has suggested that a combination of physical exercise with sensory enrichment has the strongest effect on the genesis of new neurons—predominantly in the hippocampus—and that only this combination ensures the enduring survival of the newborn cells (Kempermann et al., 1997; van Praag et al., 2005). Kattenstroth et al. (2013) have suggested that “dancing activities should be regarded as an equivalent of enriched environmental conditions for humans since they provide an individual with increased sensory, motor and cognitive demands.” Despite this encouraging statement, studies examining the effects of dance training on brain structure or function are scarce. After a 6-month dancing intervention, Kattenstroth et al. (2010) have reported significant improvements in cognitive, tactile and motor performance in participating seniors. The results of a prospective study of 469 subjects older than 75 years over a median follow-up period of 5.1 years have indicated that dancing is associated with a markedly reduced risk of dementia (Verghese et al., 2003). However, Hüfner et al. (2011) have reported reduced volumes in several brain regions including the anterior hippocampal formation and parts of the parieto-insular vestibular cortex in professional dancers and slackliners compared with non-professionals. This finding may indicate that intensive and repetitive training of the same motor skills leads in context of specialization to reduced volumes of some brain regions. In this study, we therefore design a special dance training program that constantly required the participants to learn new movement patterns (Müller et al., 2016). To assess the specific benefits of this intervention, we compared our newly designed dance program to an active rather than a passive control group, which took part in a conventional health sport fitness program in which participants typically performed repetitive physical exercises such as bicycling on an ergometer. Furthermore, because we were interested in the temporal dynamics of the interventions, we assessed the effects on brain structure and function after 6 and 18 months of training. In doing so, we sought to assess whether it is beneficial to extend interventions, because some brain regions may require more training than others, or whether there is a limit after which more training becomes detrimental. Finally, in search of a potential mechanism underlying neuroplasticity, we measured the BDNF levels in the peripheral blood. Several studies have suggested that BDNF promotes the differentiation of new neurons and synapses (Huang and Reichardt, 2001; Lessmann and Brigadski, 2009; Park and Poo, 2013; Edelmann et al., 2014). BDNF, therefore, has been proposed to be a mediator of adult neuroplasticity (Flöel et al., 2010).

## Materials and Methods

### Participants and Experimental Design

**Figure 1:**
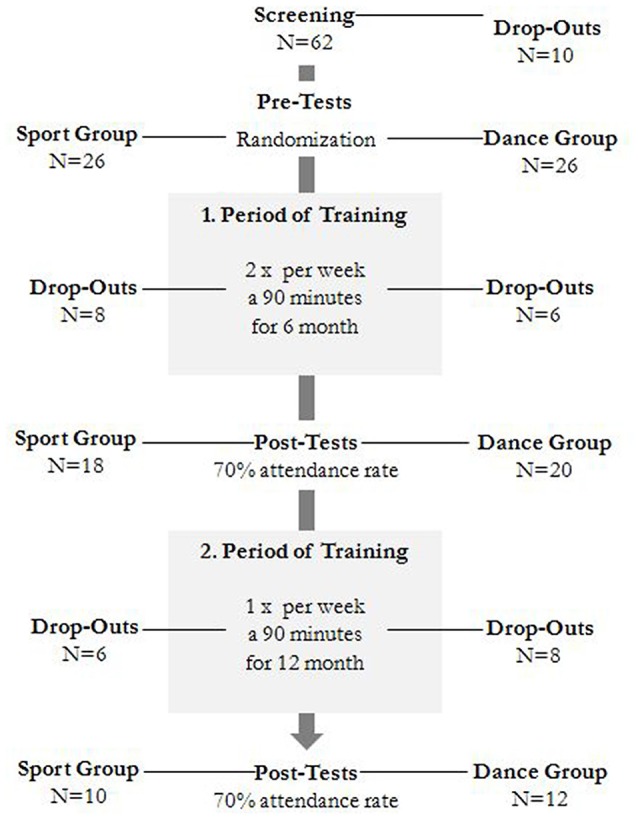
**Flow chart of participants’ recruitment**.

The study was designed as an 18-month controlled intervention. The study was approved by the ethics committee of Otto-von-Guericke University, Magdeburg, and all subjects signed a written informed and they did not receive payment for their participation. Sixty-two healthy elderly individuals (63–80 years) recruited via announcements in local newspapers were screened for the study. The exclusion criteria were claustrophobia, tinnitus, metal implants, tattoos, diabetes mellitus, depression (Beck-Depressions Inventory, BDI-II > 13), cognitive deficits (Mini-Mental State Examination, MMSE < 27), neurological diseases and regular exercising (≥1 h/week). On the basis of these criteria, 10 subjects were excluded. The remaining 52 participants were then randomly assigned to either the dance or the sport group by using the website www.randomization.com and controlling for age, MMSE status and physical fitness. Assessments were performed at baseline, after 6 and after 18 months of training (Figure 1). Twenty-two participants completed the entire intervention and all measurements. Table 1 provides detailed demographic data for these participants. No group differences regarding the demographic data were found.

**Table 1:** **Demographic information on the participants at baseline**.

Measure	Dancing group	Sport group
*N*	12	10
Age (years)	68.25 (3.91)	68.60 (2.79)
Gender (% female)	50%	40%
BMI	27.51 (3.87)	27.24 (2.94)
BDI-II	5.50 (2.94)	3.00 (3.77)
Education	15.50 (2.11)	16.40 (1.35)
MMSE	28.33 (1.07)	29.10 (0.57)

*BMI, body mass index; MMSE, Mini-Mental State Examination; BDI-II, Beck-Depressions Inventory*.

### Interventions

The interventions were separated into two periods. In the first period, the subjects trained twice per week in 90-min sessions for 6 months. For practical reasons (availability of participants and trainers), the second 12-month intervention period comprised a reduced training frequency of once per week. Both intervention programs were performed in a group context with music to control for psychosocial interactions. Conditional load was examined by recording the pulse values during the training sessions and by calculating the individual training heart rate according to Karvonen et al. (1957) with a factor 0.6 for extensive endurance training. Hence, the two training programs were comparable in terms of intensity, duration and frequency. Both were supervised by experienced instructors.

#### Dance Group

Participants in the dance group attended a newly designed training program in which they were constantly asked to learn new movement sequences. These choreographies required the coordination of different body parts (i.e., legs, arms, trunk) in space under different strain conditions (physical strain, precision, situation and time pressure). The subjects had to learn the choreographies by heart, thus imposing high demands on memory as well. The program comprised five different genres (line dance, jazz dance, rock “n” roll and square dance), which were switched after every fourth session. Over the course of the intervention, coordinative demands and time pressure were increased by introducing more complex dance moves and choreographies and by increasing the beats per minute in the music.

#### Sport Group

Participants in the sport group completed a conventional strength-endurance training program with mainly repetitive exercises and low demands in terms of whole-body coordination and memory. Each session comprised 20-min units of endurance, strength-endurance and flexibility training. The endurance training was performed on cycle ergometers. In the strength-endurance unit, alternating movements (e.g., biceps curls, squats, sit-ups) were performed, but complex whole body movements were avoided to keep coordinative demands low. The flexibility unit mainly consisted of stretching exercises.

### Outcome Measures

#### Cardiovascular Fitness

Cardiovascular fitness was assessed by the Physical Working Capacity 130 Test (PWC 130). PWC 130 is the power output (measured in watts) that a subject is able to achieve on a cycle ergometer under a heart rate of 130 bpm. For calculations, we used resting heart rate [min^−1^] and relative physical capacity [watt/kg].

#### Neuropsychological Testing

An extensive battery of neuropsychological tests were performed on the subjects. For the purpose of the current study, only the results of a verbal short- and long-term memory test, the “VLMT” (an adapted German version of the “Rey Auditory Verbal Learning Test”; Helmstaedter and Durwen, 1990) and an attention test battery (Test of Attentional Performance (TAP); Zimmermann and Fimm, 2002) are reported.

#### BDNF

Fasting blood samples were taken in the mornings of the neuropsychological assessments. From the blood samples, plasma concentrations of BDNF were determined by sandwich ELISAs (BDNF DuoSets; R&D Systems, Wiesbaden, Germany) as previously described (Schega et al., 2016).

#### MRI

MR images were acquired on a 3 Tesla Siemens MAGNETOM Verio (Syngo MR B17) using a 32-channel head coil. High-resolution T1-weighted MPRAGE sequences were acquired using a 3D magnetization-prepared rapid gradient echo imaging protocol (224 sagittal slices, voxel size: 0.8 × 0.8 × 0.8 mm^3^, TR: 2500 ms, TE: 3.47 ms, TI: 1100 ms, flip angle: 7°). The MR images were analyzed using voxel-based morphometry (VBM) implemented in SPM 12 (Welcome Department of Cognitive Neurology, London, UK). VBM is a whole-brain unbiased technique for analysis of regional gray matter volume and tissue changes (Ashburner and Friston, 2000).

Preprocessing involved gray-matter segmentation, template creation via DARTEL, spatial normalization to standardized Montreal Neurological Institute (MNI) space and smoothing with an Gaussian kernel of 8 mm full width at half maximum (FWHM).

To analyze the difference in gray matter volume changes between groups, a full-factorial design with the factors group (dance, sport) and time (0, 6 and 18 months) was applied. In the case of significant group × time interactions, *post hoc* *t*-tests between consecutive pairs of time points (0 vs. 6; 0 vs. 18; 6 vs. 18 months) were calculated separately for each group. A threshold of *p* < 0.001 (uncorrected) was applied for all analyses.

## Results

The presentation of the results is structured as follows. We first looked for a general intervention effect (factor time), then looked for differential effects of the two interventions (interaction group × time); finally, a more detailed analysis of the temporal dynamics was performed via *post hoc* pairwise comparisons.

### Cardiovascular Fitness

Cardiovascular fitness as measured by the PWC 130 (Table 2, relative physical capacity) did not differ between groups at baseline. Furthermore, the PWC 130 score did not increase significantly throughout the time course of either intervention.

**Table 2:** **Means and (SD) for fitness, cognitive functioning, BDNF plasma levels and total gray matter volume within training groups over the intervention**.

	Dance group			Sport group		
Variable	Baseline	6 months	18 months	Baseline	6 months	18 months
Relative physical capacity (Watt/kg)	1.28 (0.33)	1.19 (0.29)	1.36 (0.38)	1.19 (0.33)	1.39 (0.30)	1.21 (0.31)
Resting heart frequency (min^−1^)	77.50 (12.64)	76.08 (10.21)	73.83 (7.66)	72.00 (14.86)	69.75 (15.96)	75.00 (12.58)
VLMT early recall (points)	47.83 (10.24)	43.92 (8.29)	52.42 (6.86)	53.10 (8.00)	53.30 (8.68)	56.22 (5.19)
VLMT late recall (points)	10.25 (3.34)	9.08 (2.94)	9.90 (4.15)	12.00 (3.23)	11.70 (3.09)	14.00 (1.73)
VLMT recognition (points)	10.25 (3.08)	8.92 (3.42)	11.17 (2.21)	11.40 (3.20)	11.80 (2.86)	12.89 (1.965)
TAP flexibility reaction time (ms)	978.58 (241.15)	901.75 (288.62)	772.92 (168.97)	873.70 (234.04)	863.50 (277.63)	792.70 (162.17)
BDNF plasma	1469.57 (1038.87)	2189.59 (1116.28)	1725.83 (778.27)	1861.17 (1284.69)	2170.76 (1285.04)	1610.80 (848.59)
Gray matter volume (mm^3^)	601.95 (32.26)	597.03 (33.66)	611.27 (33.23)	593.75 (40.44)	585.89 (34.54)	602.16 (40.75)

### Neuropsychological Tests

A significant main effect of time was observed for verbal short-term memory (VLMT early recall; *F*_(2,19)_ = 6.438, *p* = 0.004, η^2^ = 0.253), verbal long-term free recall (VLMT late recall; *F*_(2,19)_ = 3.387, *p* = 0.049, η^2^ = 0.244), verbal long-term recognition (VLMT recognition; *F*_(2,19)_ = 5.352, *p* = 0.009, η^2^ = 0.220) and attention reaction time (subtest flexibility; *F*_(2,19)_ = 19.156, *p* < 0.001, η^2^ = 0.489). No significant time × group interactions emerged.

*Post hoc* pairwise comparisons showed significant improvements from baseline to 18 months and from 6 to 18 months in all three VLMT subcategories in both groups. Regarding TAP reaction times, a significant improvement was seen in the comparison of the baseline to the 18-month data in both groups.

### BDNF

**Figure 2:**
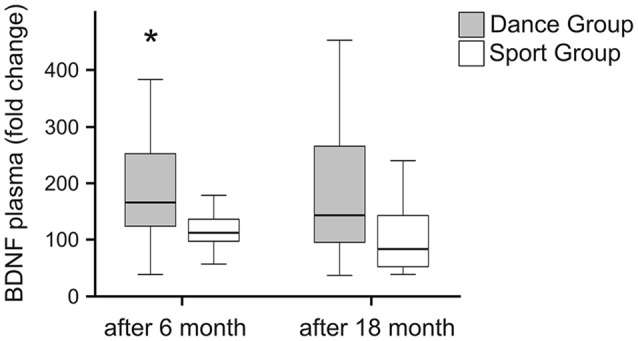
**Intraindividual changes in BDNF plasma levels after intervention.** BDNF plasma levels were analyzed in blood samples of participants performing a dancing training program or a sport training program before the onset of training, after 6 months of training and after 18 months of training. The relative increase in BDNF levels was quantified. The BDNF levels significantly increased in the dance group after 6 months of training (Mann-Whitney *U*-test, *p* < 0.004) and declined nearly to baseline of the pretreatment value after 18 months of dancing training. There was no change over the entire time course in the sport group (Friedman test, *p* = 0.319), whereas a significant change over the entire time course was observed in the dance group (Friedman test, *p* = 0.028). Box plots: minimum, 25th percentile, median, 75th percentile. *p ≤ 0.05.

Plasma levels of BDNF were analyzed in blood samples before the onset of training as well as 6 months and 18 months after the onset of training. Absolute BDNF plasma levels are summarized in Table 2. The intraindividual changes in BDNF level revealed a significant increase in the dancing group, whereas the intraindividual BDNF level remained constant after 6 months of training in the sport group. No further changes in BDNF occurred after 18 months in any group (Figure 2).

### MRI

**Figure 3:**
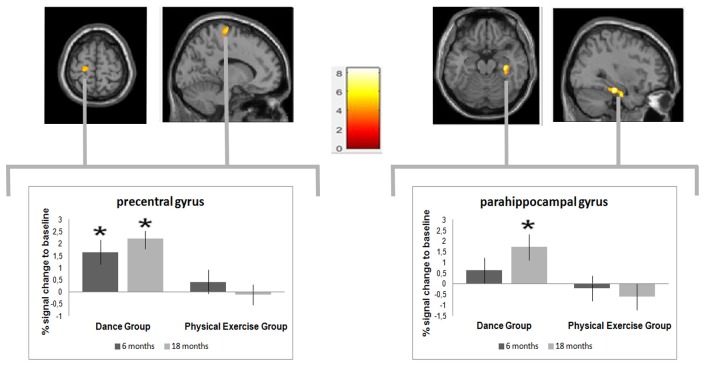
**Time by group interaction analysis, testing for greater volume changes in the dance compared with the sport group.** A significant increase in gray matter was found in the precentral gyrus (Montreal Neurological Institute (MNI)-coordinates: *x* = −16; y = −18; *z* = 77) and in the parahippocampal gyrus gyrus (MNI-coordinates: *x* = 34; *y* = −26; *z* = −20). The box plots show the relative gray matter changes in the peak voxel. *p ≤ 0.05.

A significant group × time interaction was observed in the left precentral gyrus and the right parahippocampal. *Post hoc t*-tests between the baseline and 6-month data showed a significant increase in gray matter volume in the left precentral gyrus of only the dancers. In the comparison of the baseline and the 18-month data in addition to the precentral gyrus, the dancers exhibited a significant gray matter volume increase in the right parahippocampal gyrus, which was also the only significant change in the interval from 6 to 18 months. Thus, the volume increase in the precentral gyrus emerged after 6 months and remained stable over the remaining dance training interval, whereas the change in the parahippocampal gyrus occurred during the later training interval only (Figure 3).

## Discussion

In this study, we compared the effects of participation in either a dance program or a conventional physical fitness sport program on brain function and volume in healthy seniors. The dance program was a newly designed intervention that required constantly learning new dance choreographies. The conventional sport program focused mainly on repetitive motor exercises. As a main finding, we observed that after 6 months of training, the volumes in the left precentral gyrus of the dancers had increased more than those in the sport group. After another 12 months of training, an additional volume increase was observed in the right parahippocampal gyrus of the dancers. BDNF levels increased during the first 6 months of dance training and returned to the pre-treatment values after 18 months. In the conventional sport group, a similar increase in BDNF was not evident. Because the cardiovascular fitness levels over the course of the interventions remained constant in both groups, the observed effects could not be attributed to improvements in physical fitness but instead seemed to be related to the specific features of the dance program. These features included the requirement to constantly learn new choreographies (i.e., memory), to integrate multisensory information, to coordinate the whole body and to navigate in space.

### Brain Changes

The precentral gyrus is essential for the control of voluntary motor functions. The increase in gray matter volume in the precentral gyrus in the dance group may, therefore, have been based on the complex and ever-changing movement patterns that the dancers had to perform. These movements required the simultaneous coordination of several parts of the body in different directions and adjustment to the varying rhythms of the music (polycentric and polyrhythmic). Reflecting on these complex coordination requirements, Brown et al. (2006) have reported dance-induced activations in the putamen, the primary motor cortex and the supplementary motor area (SMA), as shown by PET. Other studies have indicated an association between coordination demands (e.g., balancing, juggling) and neuroplasticity in the precentral region (Boyke et al., 2008; Taubert et al., 2010). Hence, the dance-related volume increase in this area was consistent with expectations based on the literature.

The parahippocampal gyrus is part of the outer arc of the limbic system and plays an important role in working memory and episodic memory retrieval (Pantel et al., 2003). According to Bliss and Lomo (1973), the parahippocampal gyrus constitutes the interface between memory and the experiential consciousness of the present, because it is interconnected by the perforant tract both to regions of the frontal lobe, which are associated with working memory, and to the hippocampus, the central structure in episodic memory encoding and spatial navigation. Many VBM studies have reported an age-related volume loss in parahippocampal regions (Tisserand et al., 2002). Furthermore, Echávarri et al. (2011) have suggested that parahippocampal atrophy is an early biomarker of AD. The observation that engaging in a dance program for a longer period can induce neuroplastic processes in this crucial memory region, therefore, particularly encouraging in terms of developing prevention strategies.

### Temporal Dynamics of Gray Matter Brain Plasticity

The observed volume increases in the two brain regions developed at different times. Dancing led to a volume increase in the motor areas after 6 months (see also Rehfeld et al., 2016), which remained stable during the subsequent 12 months. The dance-associated volume increase in the parahippocampal gyrus emerged later and was observed only in the 18-month data. The different temporal dynamics in the evolution of the two brain regions may be related to differences in the underlying cellular mechanisms. Animal research has suggested that angiogenesis and the formation of new dendrites (Thomas et al., 2012) occur rapidly, whereas changes in the neuropil occur much slower (Black et al., 1990). In humans, rapid increases in gray matter volume in the prefrontal regions have been observed after only 2 weeks of motor learning in younger adults (Taubert et al., 2010). To induce neuroplasticity in the hippocampus, longer training periods have been recommended (Erickson et al., 2012; Niemann et al., 2014).

Interestingly, the aforementioned volume increases from our study are in contrast to the results of Hänggi et al. (2010), which revealed that professional ballet dancers have decreased gray matter volumes in the left premotor cortex, SMA, Putamen and superior frontal gyrus and the results of Hüfner et al. (2011), which report reduced volumes in several brain regions including the anterior hippocampal formation in professional dancers and slackliners compared with non-professionals. However, these studies were cross-sectional observational studies which compared the brains of professional dancers to those of non-professionals. In addition, our dance group consisted of old aged novices. It has been shown that learning of a new skill first leads to recruitment of additional neural resources. Later, when the skill becomes more automatic, less neuronal resources are needed which may lead to volume decreases in those with long-term experience. Based on these initial conditions (novices, older adults) and our special designed dancing training program, which required constantly new learning of movement patterns, it is possible that effects of specialization induced volume decreases as reported by Hänggi et al. (2010) are not observed.

It is generally thought that motor training initially induces brain volume increases. However, prolonged training leads to automatization, which may have the opposite effects on cortical volume, because less cortical control is needed after the motor skills have been fully established (Taube, 2008). Our dance training program, therefore, was specially designed to avoid such automatization, which may explain why, at least within 18 months, no cortical volume decreases were observed in our study.

Cognitive functions also showed nonlinear development, whereby verbal memory increased only during the second training period. Regarding attention performance, significant improvements were observable after only 6 months in both groups (Rehfeld et al., 2016). These findings support prior reports regarding the beneficial effects of physical interventions on neuropsychological tests (Bamadis et al., 2014). However, in cognitive ability data, in contrast to the brain data, no group differences emerged. Others have reported superior effects of combined cognitive and physical training as opposed to single interventions (Oswald et al., 2006). We will extend our interventions further to test whether group differences in cognition might emerge at even later time points.

### Underlying Cellular and Molecular Mechanisms of Gray Matter Plasticity

Although VBM is an imaging modality that reveals volume changes in the brain, this technique does not allow causal conclusions regarding the underlying neurophysiological processes. Neurogenesis, synaptogenesis and angiogenesis are just a few of the mechanisms that have been suggested to be the basis of brain volume changes (Zatorre et al., 2012). As mediators of the effects of cardiovascular fitness on the brain, growth factors such as BDNF, insulin-like growth factor (IGF) and nerve growth factor (NGF) are being studied (Kirk-Sanchez and McGough, 2014). However, in our study, in contrast to previous ones (Erickson et al., 2011; Maass et al., 2015), no differences in cardiovascular fitness were present between groups, and the fitness levels did not change during the interventions. The latter observation was probably related to our control of the individual heart frequency, which we aimed to maintain in the aerobic zone. However, BDNF changes have also been associated with physical activity, social interaction and positive stress (Mattson, 2008), and not all studies have observed a BDNF increase after cardiovascular training (Vital et al., 2014). Finally, animal research has suggested that coordination but not endurance training induces synaptogenesis and glial changes (Black et al., 1990). Together, the named additional factors that drive BDNF secretion may have been more crucial during dancing than during fitness activities, thus explaining why only dancers showed a BDNF increase in the first 6 months. The observation that BDNF levels returned to baseline in the following 12 months while volume increases were simultaneously observed in the parahippocampal gyrus, however, indicates that there must be other factors involved in adult brain plasticity than the ones represented by BDNF levels in the peripheral blood.

Regarding neurobiological mechanisms of exercised-induced plasticity also concept of brain reserve (Satz et al., 2011) should be considered. The concept of brain reserve describe individual differences in an increased baseline adaptive neuroplasticity, which provide greater dynamic capacity for remodeling cortical circuits to different stressors (Barulli and Stern, 2013; Freret et al., 2015).

### Perspectives

The results of our study suggest that a long-term dancing intervention could be superior to repetitive physical exercise in inducing neuroplasticity in the aging human brain. We presume that this advantage is related to the multimodal nature of dancing, which combines physical, cognitive and coordinative challenges. To our knowledge, this is the first longitudinal, randomized study to recommend dancing programs as a means of preventing gray matter and cognitive decline in the elderly. Further research is needed to clarify in greater detail the temporal dynamics and the underlying neurobiological mechanisms of dance-induced neuroplasticity and whether this intervention truly has the potential to reduce the risk of neurodegenerative diseases such as Alzheimer’s.

## Author Contributions

PM designed and performed the research, analyzed the data, wrote the article. KR designed and performed the research. MS analyzed the data. AH and VL designed the research. MD: data collection, article revision. TB and JK designed the research, analyzed the data. NGM designed the research, wrote the article.

## Conflict of Interest Statement

The authors declare that the research was conducted in the absence of any commercial or financial relationships that could be construed as a potential conflict of interest.
